# Correlation of body mass index and total body fat with physical activity pattern in adolescents

**DOI:** 10.4103/0973-3930.54366

**Published:** 2009

**Authors:** A. Rajeev

**Affiliations:** Department of Epidemiology, Oman Medical College, Sohar, Sultanate of Oman, Oman

Dear Sir,

Obesity has become a worldwide problem with its share of troubles among school children even in countries like India.[1] We were encouraged to initiate an education programme for preventing and controlling obesity in school children[2,3] among the students of 5^th^ to 7^th^ divisions by the Parent-Teacher Association (PTA) of a school in the year 2004. The education programme contained topics such as the reasons for obesity, the role of diet in obesity and the need for various activities to expend the additional calories taken in the form of various junk food. The school involved had a room dedicated to indoor exercises, if some of the children found the outdoor activities unsuitable for them. The children were reported to have changed their extra-curricular behaviour as per the PTA members [Table 1] and they were encouraged to follow-up on the initial endeavour.

**Table 1:** Various activities carried out in the previous 4 weeks as reported

	Male	Female	*P*-value
Running/jogging	59	56	0.576
Walking	60	58	0.464
Dancing	7	26	0.000
Exercise	5	9	0.179
Ball games	93	76	0.031
Racket games	39	29	0.464
Cycling	71	40	0.001
Skipping	2	24	0.0001
Cricket	51	3	0.000
Climbing	22	25	0.331
Swimming	17	3	0.003
Martial Arts	9	0	0.004
Board games	7	3	0.263
Yoga	8	25	0.000

Table 2 shows the age-wise break-up of the initial cohort of 200 pupils who had relatively higher Body Mass Index (BMI) compared to their peers.[4] Since the pubertal age was nearing, we wanted to see the effects of various interventions on the growth spurt and resultant increase in prevalence of obesity in these children.

**Table 2:** The age-wise split up of Students

Age in years	Male	Female	Total	χ^2^ and *P*-value
9	16	3	19	
10	37	36	73	17.91
				P < 0.001
11	29	35	64	
12	12	32	44	
Total	94	106	200	

The initial (2004) weight, height and BMI and the change after one year of the initiative (2005) are given in Table 3. There were significant increases in all the parameters because the growth spurt was already happening in many children especially the girls. The BMI values which were somewhat different at the baseline between girls and boys equalized in the second set of measurements after one year of the initial health education.

**Table 3:** The anthropometric parameters in the first two years of study

	2004		2005	
		
	Male (N = 94)	Female (N = 106)	Male (N = 94)	Female (N = 106)
Weight	32.58 (8.01)	34.24 (7.81)	38.55 (9.40)	38.24 (8.79)
Height	140.78 (7.9)	141.03 (8.23)	148.18 (9.66)	147.63 (7.55)
Body mass index	16.26 (2.94)	17.05 (2.76)	17.36 (3.08)	17.34 (3.02)

The differences in body mass index were obviously due to the earlier growth spurt in female children. The subsequent attempt was to see if all the educational and motivational approaches derived any benefits at the end of the third year of intervention in the context of pubertal growth spurt and to start an aerobics awareness session for the students of the whole school [Table 4]. An attempt was made to see whether the overweight kids improved their extra curricular activities. The change scores of weight, height and BMI only could be correlated with the activity levels of the children and results are presented in Table 5.

**Table 4:** The anthropometric parameters in the 4^th^ year of study as compared to the baseline values

	Male (N = 94)		Female (N = 104)	
		
	2004	2007	2004	2007
Weight	32.58 (8.01)	42.45 (8.44)	34.24 (7.81)	41.71 (10.10)
Height	140.78 (7.9)	150.52 (7.91)	141.03 (8.23)	151.21 (10.92)
Body mass index	16.26 (2.94)	18.64 (2.96)	17.05 (2.76)	18.13 (3.52)

**Table 5:** Correlation of activity frequency with the anthropometric parameters

	Strenuous activities	Moderate activities	Mild activities
Weight 2005	−.151	−.208**	−.193*
Height 2005	−.231**	−.315**	−.153
Body mass index 2005	−.039	−.065	−.151

* Other non-significant variables not snown in tne table

** Statistically significant

For measuring the activity levels of students the Godin Leisure-Time Exercise Questionnaire was given to all the students of the study to measure the frequency at which they indulged in various grades of physical activity during their leisure hours for more than 15 minutes. Some activities were classified as strenuous, some as moderate and some mild activities. The number of times they indulged in such activities in the past week was queried and the final score was calculated based on the equation: Weekly leisure activity score = (9 × Strenuous) + (5 × Moderate) + (3 × Mild).

The values obtained and also calculated from the questionnaire were correlated with BMI and other parameters. The bivariate correlations of the exercise pattern are shown in Table 5. The taller and heavier children were found generally to engage in more strenuous kind of activities. The ones with larger weight were engaged in moderate activities. More obese would stick to milder activities. In the follow-up phase of the study they were again asked about the frequency of strenuous activities in broader terms. They were also asked about the time they allocated to doing various physical activities.

In multi-variate analysis, BMI was shown to be correlated only with time spent on various activities as also with the frequency of the mild category of activities than a vigorous style of extra-curricular functioning [Table 6]. The total score for Godin Activity questionnaire also correlated with BMI very weakly. The mixed pattern of results was, possibly, as the result of increase in muscle mass of the children owing to the pubertal growth spurt as well as physical activities spurred by the education. Body Mass Index, obviously, was correlating with inactivity than with activity. The results were not motivating enough to induce the parents to get involved in promoting the children to engage in more aerobic exercise activities with some supportive evidence and to take up aerobics lessons for the whole school.

**Table 6:** Regression of BMI in the year 2005 on various activity parameters

Variable*	Type III Sum of Squares	df	Mean Square	F	*P*
Intercept	8573.703	1	8573.703	343.316	0.000**
Frequency of mild activity per week	153.983	1	153.983	6.256	0.014**
Time spent in the last 4 weeks on physical activity	104.548	1	104.548	4.247	0.041**
Total Godin Physical Activity Score	80.976	1	80.976	3.290	0.072
Age	150.124	3	50.041	2.033	0.112
Frequency of Strenuous activity per week	60.357	1	60.357	2.452	0.120

However, the second year of interventions were also a stepped-up version of the first year programme as we wanted to demonstrate to the children the effects of gaining too much of weight. These comprised of measurement of their Blood Pressure (BP) and many other anthropometric measurements. [Table 7] The BP measurement was done in the high BMI (> 85^th^ centile) category using an electronic (wrist) BP apparatus which was enough to gather attention of the students. There were no differences in these values either by age categories or gender categories (Statistics not shown). The higher BP values were double-checked with the same equipment and a mercury manometer subsequently. A paediatrician evaluated the children to classify the BP values based on individuals' height percentiles and to rule out secondary causes for high values.

**Table 7:** Some more anthropometric measurements of the study group

	Mid arm circumference	Skin-fold thickness	Systolic BP	Diastolic BP
Males	23.55 (2.99)	19.29 (7.81)	113.90 (10.77)	72.08 (10.78)
Females	22.34 (3.32)	20.75 (6.70)	117.30 (12.28)	73.05 (8.38)
Total	22.90 (3.20)	20.08 (7.19)	115.70 (11.63)	72.58 (9.57)

Simultaneously, bio-impedance measurements were done in 92 high BMI children and various relevant parameters were derived[5] as shown in Table 8. There was significant increase in the Total Body Water and Fat-Free Mass related to age but not for gender (Statistics not shown). The mid-arm circumference and skinfold measurement along with weight and height were used to generate predictive equations for the Total Body Fat (TBF in kg). The predictive equations derived from various anthropometric variables like midarm circumference and skinfold thickness did not correlate significantly with any of the activity patterns and were dropped from the study design.

**Table 8:** The age-wise analysis of BMI and Body Fat percent of an obese subgroup according to gender in the year 2005

Age in	Males (N = 94)		Females (N = 106)	
		
years	BMI	Body Fat%	BMI	Body Fat%
10	-	-	19.13 (0.57)	13.50 (2.80)
11	18.23 (3.51)	9.11 (4.86)	18.46 (3.07)	11.27 (7.23)
12	21.85 (3.36)	15.61 (6.73)	18.50 (3.62)	12.06 (5.33)
13	20.55 (2.86)	19.29 (14.74)	19.76 (2.42)	18.35 (2.57)
Total	20.27 (3.49)	14.49 (9.74)	18.71 (2.88)	17.34 (3.03)

Only TBF assessed directly by the bio-impedance equipment correlated with the detailed exercise assessment [Figure 1]. BMI did not correlate with the activity patterns (except with mild activities in multi-variate analysis) and one already knew that it cannot differentiate between the increase in growth due to muscle mass or fat mass.[6] This was a significant finding in that the physical activity, or rather, inactivity has been shown to correlate with BMI in many of the previous studies. Our aim in promoting physical activity shall not be to curtail growth, rather, to reduce fat accumulation. Our findings also demonstrated that moderate activities (not exhausting) e.g., fast walking, baseball, tennis, easy bicycling, volleyball, badminton, easy swimming, popular and folk dancing were enough to reduce fat accumulation [Figure 1].

**Figure 1:**
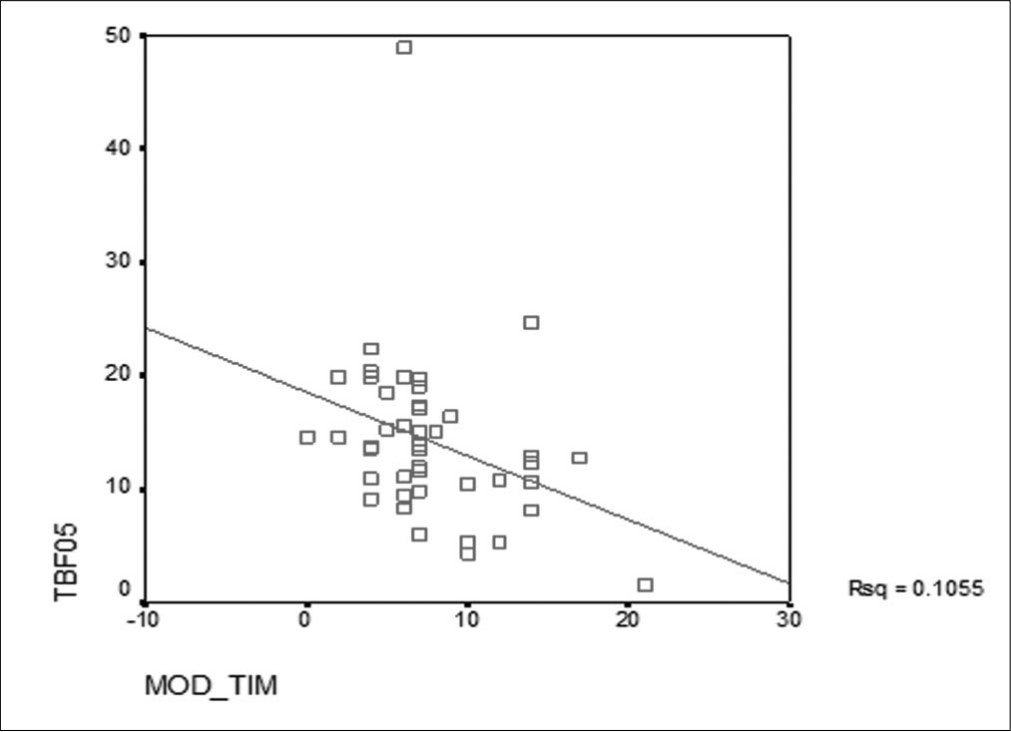
Correlation between Frequency of Moderate Activities (Mod_tim) and Total Body Fat (TBF05 - NHANES)

The strenuous activities also reduced the TBF though not significantly as the moderate kind of activities. We could conclude that the best form of exercise which suited one and all would be aerobic dancing which was easy for all groups concerned, required no additional skills, routines are full of fun and no additional equipment was required[7] (Public address system in the school could be used for the music). The teachers were trained in the activity such that the programme is self-supporting and required no additional manpower. Strenuous activities could motivate only the physically stronger subset and hence were not attractive to most of the average students.

**Table 9:** The frequency of leisure activity as per the various categories of BMI

BMI	Grades of Activity			Total
		
Percentile	Frequently	Sometimes	Rarely	
Upto 10th	0 (0)	28 (27.7)	7 (9.3)	35 (19.6)
10th – 50th	1 (33.3)	43 (42.6)	36 (48.0)	80 (44.7)
>50th	2 (66.7)	30 (29.7)	32 (42.7)	64 (35.8)
Total	3 (100)	101 (100)	75 (100)	179* (100)

* 21 missing data: χ^2^ = 11.25, df = 4, *P* = 0.024

All the more, the strenuous activities were not popular with the high BMI group.

Despite selective non-response on BMI, reliance on self-reports and lack of equipment for continuous monitoring of total body fat, the associations between activities and overweight were evident. Pubertal growth spurt alone cannot explain the increase in obesity as also age which are known variables associated with increase in BMI.[8] We have concluded that Total Body Fat Mass should have been the measure of success of strategies for prevention of overweight, rather than, Body Mass Index. However, the non-availability of costly equipment prevents such objective measurements in routine school health interventions.

## References

[1] Kaur S, Dwivedi SN, Lakshmy R, Kapil U (2008). Prevalence of overweight and obesity amongst school children in Delhi, India. Asia Pac J Clin Nutr.

[2] Jones RA, Okely AD, Collins CE, Morgan PJ, Steele JR, Warren JM (2007). The HIKCUPS Trial: A multi-site randomized controlled trial of a combined physical activity skill-development and dietary modification program in overweight and obese children. BMC Public Health.

[3] te Velde SJ, De Bourdeaudhuij I, Thorsdottir I, Rasmussen M, Hagströmer M, Klepp KI (2007). Patterns in sedentary and exercise behavior and associations with overweight in 9-14-year-old boys and girls - a cross-sectional study. BMC Public Health.

[4] WHO (World Health Organisation) International Association for the Study of Obesity (IASO) and International Obesity task Force (IOTF). 2000. The Asia-Pacific Perspective: Redefining Obesity and its treatment.

[5] Demerath EW, Guo SS, Chumlea WC, Towne B, Roche AF, Siervogel RM (2002). Body Composition estimates from NHANES III bioimpedance data. International Journal of Obesity.

[6] Banerjee I, Ghia N, Bandopadhyay S, Sayed HN, Mukherjee D (2007). Body Mass Index in Bengali Adolescents. Indian Pediatr.

[7] Rosemary F (1996). Dance for Health. Improving Fitness in African Amercian and Hispanic Adolescents. Public Health Rep.

[8] Banerjee I, Chakraborty S, Bhattacharyya NG, Bandyopadhyay S, Saiyed HN (2007). A cohort study of correlation between Body Mass Index and age at menarche in healthy Bengali girls. J Indian Med Assoc.

